# Chemoradiotherapy Is Inferior to Chemotherapy Alone in Adjuvant Setting for Signet Ring Cell Containing Gastric Cancer

**DOI:** 10.3389/fonc.2020.570268

**Published:** 2020-11-26

**Authors:** Yue-Ting Zhu, Xin-Zu Chen, Ye Chen, Yu-Wen Zhou, Lian-Sha Tang, De-Yun Luo, Qiu Li, Meng Qiu, Xin Wang, Dan Cao, Yu Yang, Ya-Li Shen, Zhi-Ping Li, Feng Bi, Ji-Yan Liu, Hong-Feng Gou

**Affiliations:** ^1^ Department of Biotherapy, Cancer Center, West China Hospital, Sichuan University, Chengdu, China; ^2^ Department of Gastrointestinal Surgery and Laboratory of Gastric Cancer, State Key Laboratory of Biotherapy, West China Hospital, Collaborative Innovation Center for Biotherapy, Sichuan University, Chengdu, China; ^3^ Department of Abdominal Cancer, Cancer Center, West China Hospital, Sichuan University, Chengdu, China

**Keywords:** signet ring cell carcinoma, gastric cancer, adjuvant therapy, chemotherapy, chemoradiotherapy

## Abstract

**Background:**

Signet ring cell containing gastric cancer (SRCGC) is a rare subtype of gastric cancer, and its adjuvant therapy is based on general gastric cancer. However, the effectiveness of radiotherapy for those SRCGC patients remains unknown.

**Purpose:**

The purpose of the study was to analyze whether the addition of radiotherapy to adjuvant chemotherapy (CT) can benefit survival in resected SRCGC patients.

**Methods:**

Patients with SRCGC, who underwent D2 gastrectomy followed by adjuvant chemotherapy or chemoradiotherapy (CRT), were retrospectively collected. According to the proportion of signet ring cells, patients were histologically classified as pure SRCGC (pSRCGC) containing 100% of signet ring cells, mixed SRCGC (mSRCGC) containing >50% of signet ring cells, and contaminated SRCGC (cSRCGC) containing <50% of signet ring cells. Among the 272 patients, 156 were treated by CT alone and 116 by CRT. The primary endpoint was 3-year overall survival rate (3-year OS rate).

**Results:**

With a median follow-up of 80.5 months, the 3-year OS rate was significantly higher in the CT group (70.5% vs. 58.6%, HR = 0.633, *P* = 0.017) compared with CRT group. Three independent characteristics were predictive of a poor overall survival: CRT treatment (*P* = 0.019), tumor size ≥5 cm (*P* < 0.001), and the presence of vessel invasion (*P* = 0.009). Subgroup analyses showed CRT significantly impaired prognosis in SRCGC patients in the cSRCGC subset, as well as lesions located in lower-middle sites, subtotal gastrectomy, male, <60 year, and no vessel invasion. Peritoneal was the most common recurrence site in SRCGC patients. The adverse events leukopenia and neutropenia were more common in the CRT group (*P* = 0.007).

**Conclusions:**

Adjuvant chemoradiotherapy was associated with poor survival compared with adjuvant chemotherapy in SRCGC patients with D2 gastrectomy.

## Introduction

Gastric cancer (GC) is the fifth most common cancer and the third leading cause of cancer-related death worldwide, with an estimated 783,000 deaths in GLOBOCAN 2018 (1). GC is a heterogeneous disease with various histological classifications. According to the World Health Organization (WHO) classification, specimen composed of more than 50% signet ring cells is histologically defined as signet ring cell carcinoma (2). Almost all signet ring cell containing gastric cancers (SRCGCs) were diffuse type by the Lauren classification (3). The incidence of SRCGC increased 10-fold between the 1970s and 2000s, mainly in Western countries (4), varying from 15.1% to 34.9% of gastric cancer in recent researches (5–7). Moreover, SRCGC has attracted more attention in recent years (8). Patients with SRCGC tend to be the younger and female, and the tumor is usually in the middle-third part of stomach (7, 9–11). Furthermore, SRCGC is associated with more advanced diseases, a higher histological grade (9, 11), and worse survival outcome than non-SRCGC, due to a higher rate of peritoneal carcinomatosis and lymph node invasion, and a lower rate of curative resection and chemoresistance (11–14).

Perioperative chemotherapy became a standard treatment for local advanced resectable gastric cancer in Western countries (15, 16). In a retrospective study of 924 resected SRCGC patients, perioperative chemotherapy was associated with a significantly impaired prognosis (6). As for adjuvant therapy for gastric cancer patients, the ARTIST and the ACTS-GC trials proved that adjuvant chemotherapy was a standard of management for D2-resected GC patients (17–19). The Intergroup 0116 (INT-0116) trial demonstrated a strong and persistent benefit from adjuvant chemoradiotherapy for D1-resected GC (20). However, the ARTIST trial noted that the addition of radiotherapy to standard chemotherapy did not signiﬁcantly reduce the rate of recurrence in D2-resected GC patients (21). Thus the effect of radiotherapy is still controversial in certain GC patients. As a subset of GC, SRCGC was found to have chemoresistance (10, 12, 13) and might not benefit from preoperative chemotherapy (6, 7). However, data of adjuvant therapy for SRCGC were rare and there were no prospective studies of adjuvant treatment on resected SRCGC only. It was supposed that primary resection should be proposed for patients with SRCGC and followed by adjuvant chemotherapy (CT) or chemoradiotherapy (CRT). But the optimal adjuvant treatment strategy for resected SRCs is still pending.

The purpose of this retrospective study was to confirm whether the addition of radiotherapy to adjuvant chemotherapy could benefit survival in patients with radically resected SRCGCs. We analyzed the overall survival for SRCGC patients in correlation with adjuvant CT and CRT. We also explored the characteristics related to poor prognosis and the pattern of recurrence in the SRCGC population. We hypothesized that SRC status may serve as a potential indicator for adjuvant treatment, therefore a tailored adjuvant treatment should be considered for patients with SRCGC.

## Materials and Methods

### Patient Collection

The medical records of patients were retrospectively collected in a central teaching hospital (West China Hospital, Sichuan University) between August 2007 and December 2014. This study was based on the Surgical Gastric Cancer Patient Registry of West China Hospital (id: WCH-SGCPR-2019-01) (22). The inclusion criteria were (1) histologically confirmed GC containing signet ring cell, regardless of the proportion of signet ring cells; (2) underwent D2 or D2+ gastrectomy with the intention of R0 resection; (3) received adjuvant systematic chemotherapy or chemoradiotherapy; 4) clinicopathological TNM stage of Ib–IIIc; and (5) no limitation on sex, age, and ethnicity. The exclusion criteria were (1) received neoadjuvant therapy; (2) R1–R2 resection; (3) double primary tumors; (4) distant metastasis; (5) recurrence; (6) received chemotherapy fewer than two cycles; and (7) other than adenocarcinoma.

### Surgery

All the patients underwent operations at West China Hospital. Distal or total gastrectomy was performed based on the location of the tumor, and a standard D2 or D2+ lymphadenectomy was generally performed according to the Japanese Gastric Cancer Treatment Guidelines (23). There was no limitation on the pattern of digestive tract reconstruction, Billroth-2 gastrojejunostomy, as well as Roux-en-Y gastrojejunostomy or Billroth-1 gastroduodenostomy; Roux-en-Y esophagojejunostomy, as well as with jejunal pouch were also accepted.

### Pathology

Tumor staging was assessed according to the American Joint Committee on Cancer/Union International Control Center TNM Staging Manual, 7th edition (24). According to WHO classification, signet ring cell gastric cancer is defined as a predominant component (>50% signet ring cells) of isolated carcinoma cells with intracellular mucin (2). However, in the present study, we analyzed those so-called “signet ring cell containing gastric cancer (SRCGC),” with the intention to investigate the influence of different signet ring cell proportion. We histologically divided the patients into three SRC statuses: pure SRCGC (pSRCGC), containing 100% of signet ring cells; mixed SRCGC (mSRCGC), containing >50% of signet ring cells; and contaminated SRCGC (cSRCGC), containing ≤50% of signet ring cells.

### Adjuvant Chemotherapy

Criteria for patients receiving adjuvant therapy are based on the NCCN Guidelines, including stage IB with high-risk factors, stages II and stage III. Patients were administered adjuvant treatment postoperative 3–8 weeks. The following primary chemotherapy schemes were accepted in our study: (1) S-1mono-regimen [body-surface area (BSA) <1.25 m^2^, 80 mg daily; BSA ≥1.25 m^2^ but <1.5 m^2^, 100 mg daily; BSA≥1.5 m^2^, 120 mg daily, d1-28, every 6 weeks]; (2) mFOLFOX6 (oxaliplatin 85 mg/m^2^, d1; CF 400 mg/m^2^, 2h, d1; 5-fluorouracil 400 mg/m^2^, iv, d1, and 2400 mg/m^2^, civ 48 h, every 2 weeks); (3) SOX (S-1 40 mg/m^2^/day, d1-14; oxaliplatin 130 mg/m^2^, d1, every 3 weeks). A less common regimen DCF (docetaxel 75 mg/m^2^, d1, cisplatin 20 mg/m^2^, d1, CF 200mg/m^2^, d1, 5-Fu 400 mg/m^2^, iv, d1 and 600 mg/m^2^, civ 48h, every 3 weeks) was also included. Among them, 29 patients received a single regimen, 235 patients received a double-agent combination, and 8 patients received triple-drug chemotherapy.

### Adjuvant Chemoradiotherapy

For postoperative chemoradiotherapy, patients received one cycle of adjuvant FOLFOX, SOX, S-1 mono-regimen, or DCF before starting radiotherapy. The 3D-CRT or IMRT technique was selected by the physician according to the complexity of the target volume and the organs at risk (OAR). Patients received CT simulation using helical CT scan and were treated in a supine position. The criterion of clinical target volume (CTV) was the gastric bed, anastomoses and stumps, and the draining lymph nodes. The planning target volume (PTV) comprised a 1.0 cm margin around the CTV. A total irradiation dose of 50.4 Gy was administered in 28 fractions of 1.8 Gy, 5 days per week. Dose constraints of critical organs were as follows: spinal cord Dmax < 40 Gy; liver V30 < 30%; two-thirds of one kidney less than 18 Gy or 30% of each kidney volume of each kidney less than 25 Gy. During the process of radiotherapy, S-1 (40 mg/m^2^/day) was orally given twice daily from day 1 to 5 per week. Two or four weeks after the completion of radiotherapy, additional cycles of regimen were given.

### Follow-Up and Outcome Measure

Follow-up lasted until June 30, 2018. The toxicity, survival status, follow-up duration, and loss were recorded. The primary endpoint was 3-year overall survival rate (3-year OS rate), referred to as the proportion of resected SRCGC patients who were alive 3 years after the primary surgery date. Treatment toxicity was graded according to the National Cancer Institute Common Terminology Criteria for Adverse Events (CTCAE, version 3.0) (25).

### Ethics

The collection of medical information for the surgical gastric cancer patients was approved by the Biomedical Ethical Committee of West China Hospital, Sichuan University. The participants were not required to sign written informed consent in this retrospective study. However, the records were anonymized and de-identified before analyses. The study complied with the World Medical Association Declaration of Helsinki regarding the ethical conduct of research involving human subjects.

### Statistics

Statistical analysis was performed using the Statistical Package for Social Science (SPSS), version 23.0. In the baseline comparisons, the ranked variables were compared by the Mann-Whitney U test, while continuous variables were compared by the Mann-Whitney U test or one-way ANOVA test, where applicable. Categorical variables were compared by Pearson’s chi-square test or Fisher’s exact test. The survival rates were calculated by the Kaplan-Meier method, median survival times (MST) were not reached, and the 3-year OS rate was expressed. Univariate survival analyses were performed by the log-rank test. Multivariable analysis of prognostic factors was conducted by the Cox proportional hazards model. Hazard ratios (HRs) with 95% confidence intervals (CIs) were estimated. Cox models in multivariate analyses were adjusted for clinicopathologic features, and surgical and adjuvant treatment, without selection procedure. *P* < 0.05 was considered to be statistically significant.

## Results

### Patient Characteristics

**Figure 1:**
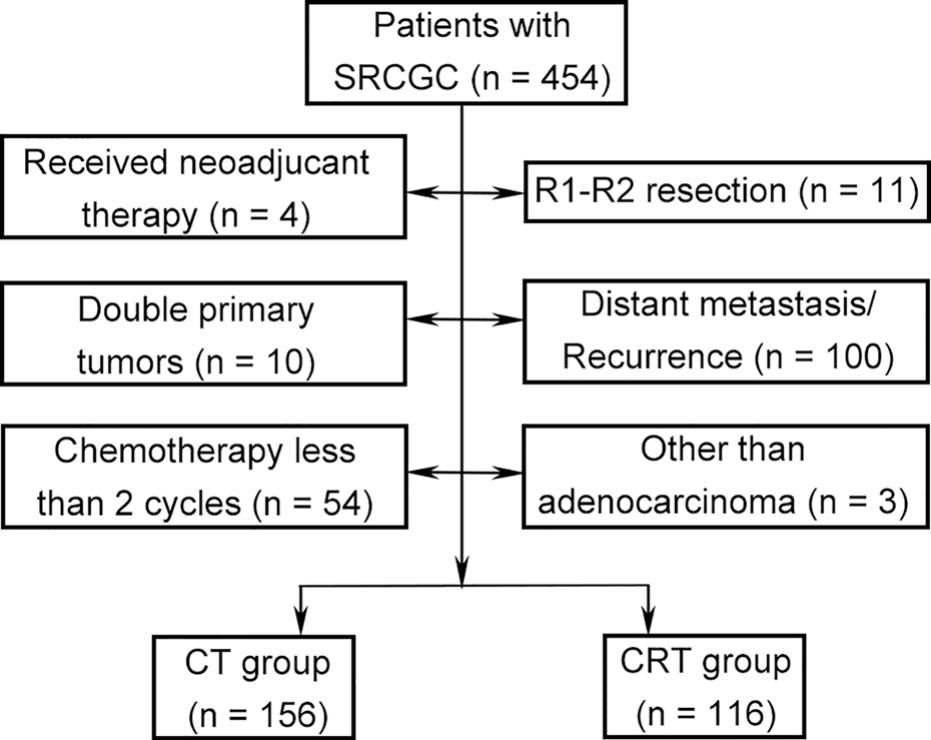
Flow chart. SRCGC, Signet ring cell containing gastric cancer; CT, chemotherapy; CRT, chemoradiotherapy.

A total of 272 patients met the inclusion criteria between August 2007 and December 2014 (**Figure 1**). Data on gender, age, tumor location, gastrectomy, tumor size, vessel invasion, perineural invasion, tumor–lymph node–metastasis (TNM) classification, T category, N category, SRC status, and adjuvant therapy strategies were collected for analysis. Baseline characteristics were summarized in **Table 1**. Among the participants, 123 (45.2%), 99 (36.4%), and 50 (18.4%) were diagnosed as cSRCGC, mSRCGC, and pSRCGC, respectively. Patients were divided into the chemotherapy (CT) group or chemoradiotherapy (CRT) group according to their adjuvant treatment strategies. There were 156 patients in the CT group and 116 in the CRT group. Patients in CT group tend to have earlier N category than those in the CRT group, *p* = 0.001.

**Table 1:** Patient characteristics (*N* = 272).

Variables	Total*N* = 272 (%)	CT group*N* = 156 (%)	CRT group*N* = 116 (%)	*P*
Gender				0.710
Male	163 (59.9)	92 (59.0)	71 (61.2)	
Female	109 (40.1)	64 (41.0)	45 (38.8)	
Age				0.347
<60	213(78.3)	119 (76.3)	94 (81.0)	
≥60	59 (21.7)	37 (23.7)	22 (19.0)	
Tumor location				0.321
Cardia/GEJ	25 (9.2)	12 (7.7)	13 (11.2)	
Non-cardia/GEJ	247 (90.8)	144 (92.3)	103 (88.8)	
Gastrectomy				0.283
Subtotal	174 (64.0)	104 (66.7)	70 (60.3)	
Total	98 (36.0)	52 (33.3)	46 (40.0)	
Tumor size				0.208
<5 cm	141 (51.8)	86 (55.1)	55 (47.4)	
≥5 cm	131 (48.2)	70 (44.9)	61 (52.6)	
Vessel invasion				0.396
No	204 (75)	114 (73.1)	90 (77.6)	
Yes	68 (25)	42 (26.9)	26 (22.4)	
Perineural invasion				0.748
No	202 (74.3)	117 (75.0)	85(73.2)	
Yes	70 (25.7)	39 (25.0)	31(26.7)	
TNM stage				0.066
I	20 (7.4)	15 (9.6)	5 (4.3)	
II	65 (23.9)	42 (26.9)	23 (19.8)	
III	187 (68.8)	99 (63.5)	88 (75.9)	
T category				0.552
T1	27 (9.9)	19 (12.2)	8 (6.9)	
T2	41 (15.1)	23 (14.7)	18 (15.5)	
T3	76 (27.9)	43 (27.6)	33 (28.4)	
T4	128 (47.1)	71 (45.5)	57 (49.1)	
N category				0.001
N0	25 (8.8)	23 (14.7)	2 (1.7)	
N1	45 (16.5)	27 (7.3)	18 (15.5)	
N2	66 (24.2)	35 (22.4)	31 (26.7)	
N3	136 (50.4)	71 (45.5)	65 (56.0)	
SRC status				0.573
cSRCGC	123 (45.2)	69 (44.2)	54 (46.6)	
mSRCGC	99 (36.4)	55 (35.3)	44 (37.9)	
pSRCGC	50 (18.4)	32 (20.5)	18 (15.5)	

CT, chemotherapy; CRT, chemoradiotherapy; cSRCGC, contaminated signet ring cell containing gastric cancer; mSRCGC, mixed signet ring cell containing gastric cancer; pSRCGC, pure signet ring cell containing gastric cancer.

### Recurrence

By the end of the follow-up date (June 30, 2018), 165 (60.7%) patients had recorded disease free survival (DFS), and 88 (53.3%) of them had local recurrence and metastases. To compare the pattern of recurrence more accurately in the different groups, we calculated the rates and sites of recurrence in those patients who had known DFS (**Table 2**). The frequencies of recurrence were comparable in the pSRCGC, mSRCGC, and cSRCGC groups (45.5% vs. 46.0% vs. 47.8%, *p* = 0.9671). In the whole cohort, the most common site of recurrence was peritoneal (22.4%), followed by lymph node (21.2%), liver (5.5%), and other sites (11.5%) (including remnant stomach, lung, gallbladder, ovary, and bone). Overall, there were no significant different sites of recurrence in the three groups (*p* = 0.0690). The median time to recurrence was 19.0 months, and no significant difference was found in time to recurrence in the three groups (21.8 months vs. 17.5 months vs. 19.0 months, *p* = 0.6724).

**Table 2:** Pattern of recurrence (*N* = 165).

Variables	Total*N* = 165 (%)	pSRCGC*N* = 33 (%)	mSRCGC*N* = 63 (%)	cSRCGC*N* = 69 (%)	*P*
Recurrence					0.9671
No	77 (46.7)	15 (45.5)	29 (46.0)	33 (47.8)	
Yes	88 (53.3)	18 (54.5)	34 (54.0)	36 (52.2)	
Sites of recurrence					0.0690
Peritoneal	37 (22.4)	8 (24.2)	14 (22.2)	15 (21.7)	
Lymph nodes^#^	35 (21.2)	9 (27.3)	15 (23.8)	11 (15.9)	
Liver	9 (5.5)	0	1 (1.6)	8 (11.6)	
Other sites*	19 (11.5)	3 (9.1)	10 (15.9)	6 (8.7)	
Median time to recurrence (months)	19.0	21.8	17.5	19.0	0.6724
[range min–max]	[1–117.5]	[3–108.5]	[2–97.5]	[1–117.5]	

^#^Some patients had both nodal and extranodal sites of recurrence.

*Other sites including remnant stomach, lung, gallbladder, ovary, and bone.

### Toxicity

The hematologic toxicities were gathered and are shown in **Table 3**. The most frequent grade 3 or 4 adverse events were leukopenia (13.6%), anemia (11.8%), thrombocytopenia (9.6%), and neutropenia (9.6%). Grade 3/4 leukopenia (19.0% vs. 9.6%, *P* = 0.026), leukopenia with any grade (74.1% vs. 57.7%, *P* = 0.005), and total neutropenia (65.5% vs. 51.3%, *P* = 0.019) were more common in the CRT group than in the CT group.

**Table 3:** Hematologic toxicity (NCI-CTCAE v3.0)* (*N* = 272).

	CT group (*N* = 156)		CRT group (*N* = 116)		*P*	
Toxicity	All grades *N* (%)	Grade III/IV *N* (%)	All grades *N* (%)	Grade III/IV *N* (%)	All grades	Grade III/IV
Leukopenia	90 (57.7)	15 (9.6)	86 (74.1)	22 (19.0)	0.005	0.026
Anemia	118 (75.6)	20 (12.8)	86 (74.1)	12 (10.3)	0.777	0.531
Thrombocytopenia	71 (45.5)	12 (7.7)	60 (51.7)	14 (12.1)	0.311	0.225
Neutropenia	80 (51.3)	13 (8.3)	76 (65.5)	13 (11.2)	0.019	0.425
Elevated AST or ALT level	69 (44.2)	6 (3.8)	54 (46.6)	5 (4.3)	0.704	0.848

*All adverse events were graded according to National Cancer Institute Common Terminology Criteria for Adverse Events (CTCAE version 3.0).

### Survival Outcomes

**Figure 2:**
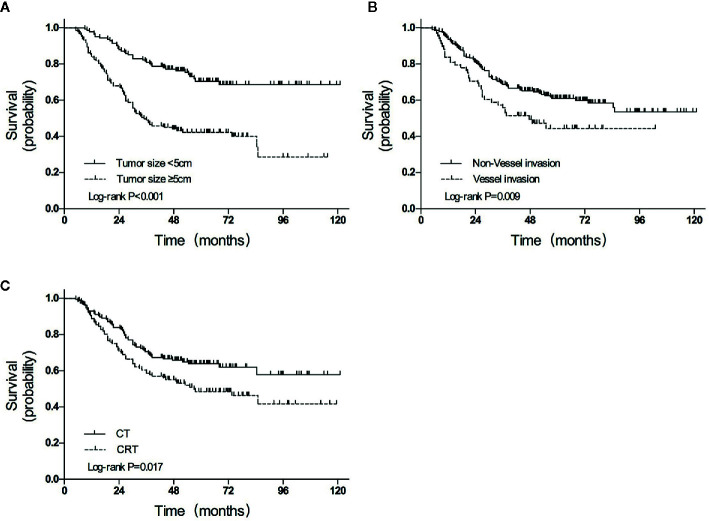
Overall survival curves for patients grouped according to **(A)** treatment, **(B)** tumor size, and **(C)** vessel invasion. CT, chemotherapy; CRT, chemoradiotherapy.

The median follow-up duration was 85.0 months (range 5.0–121.0 months), except for 8 patients with inadequate follow-up (3 in the CRT group and 5 in the CT group). A total of 108 (39.7%) of the 272 participants had died by the end of data accumulation on June 30, 2018. The 3-year OS was higher in the CT group than in the CRT group (70.5% vs. 58.6%, HR = 0.633, *P* = 0.017; **Figure 2A**).

In univariate analyses, several factors were statistically associated with poor survival: advanced TNM stage (*P* < 0.001), advanced T category (*P* < 0.001), advanced N category (*P* < 0.001), tumor size ≥5 cm (*P* < 0.001), total gastrectomy (*P* = 0.007), vessel invasion (*P* = 0.013), and CRT treatment (*P* = 0.017).

Variables with *P* values of <0.05 in the univariate analysis were included in the multivariate analysis (**Table 4**). Three independent variables were predictive of a poor outcome: CRT treatment (*P* = 0.019), tumor size ≥5 cm (*P* < 0.001), and the presence of vessel invasion (*P* = 0.009) (**Figures 2A–C**).

**Table 4:** Survival prediction by multivariate analysis of variables for patients with gastric SRC.

Variables	Total	No. events	Hazard ratio	95%CI	*P*
Gender					
Male	163	68			
Female	109	49	0.946	0.638–1.405	0.784
Age					
<60	213	88			
≥60	59	29	1.245	0.783–1.979	0.354
Tumor location					
Cardia/GEJ	25	11			
Non-cardia/GEJ	247	106	0.670	0.340–1.320	0.247
Gastrectomy					
Subtotal	174	65			
Total	98	52	0.364	0.555–1.241	0.830
Tumor size					
<5 cm	141	39			
≥5 cm	131	78	2.281	1.487–3.499	<0.001
Vessel invasion					
No	204	81			
Yes	68	36	1.740	1.148–2.639	0.009
T category					
T1,T2	68	15			
T3,T4	204	102	0.564	0.301–1.056	0.074
N category					
N0,N1	70	20			
N2,N3	202	97	0.932	0.547–1.591	0.797
SRC status					
cSRCGC	123	48			
mSRCGC	99	48	1.243	0.721–2.142	0.434
pSRCGC	50	21	1.446	0.959–2.181	0.078
Treatment					
CT	156	57			
CRT	116	60	1.574	1.079–2.295	0.019

### Subgroup Analysis

**Figure 3:**
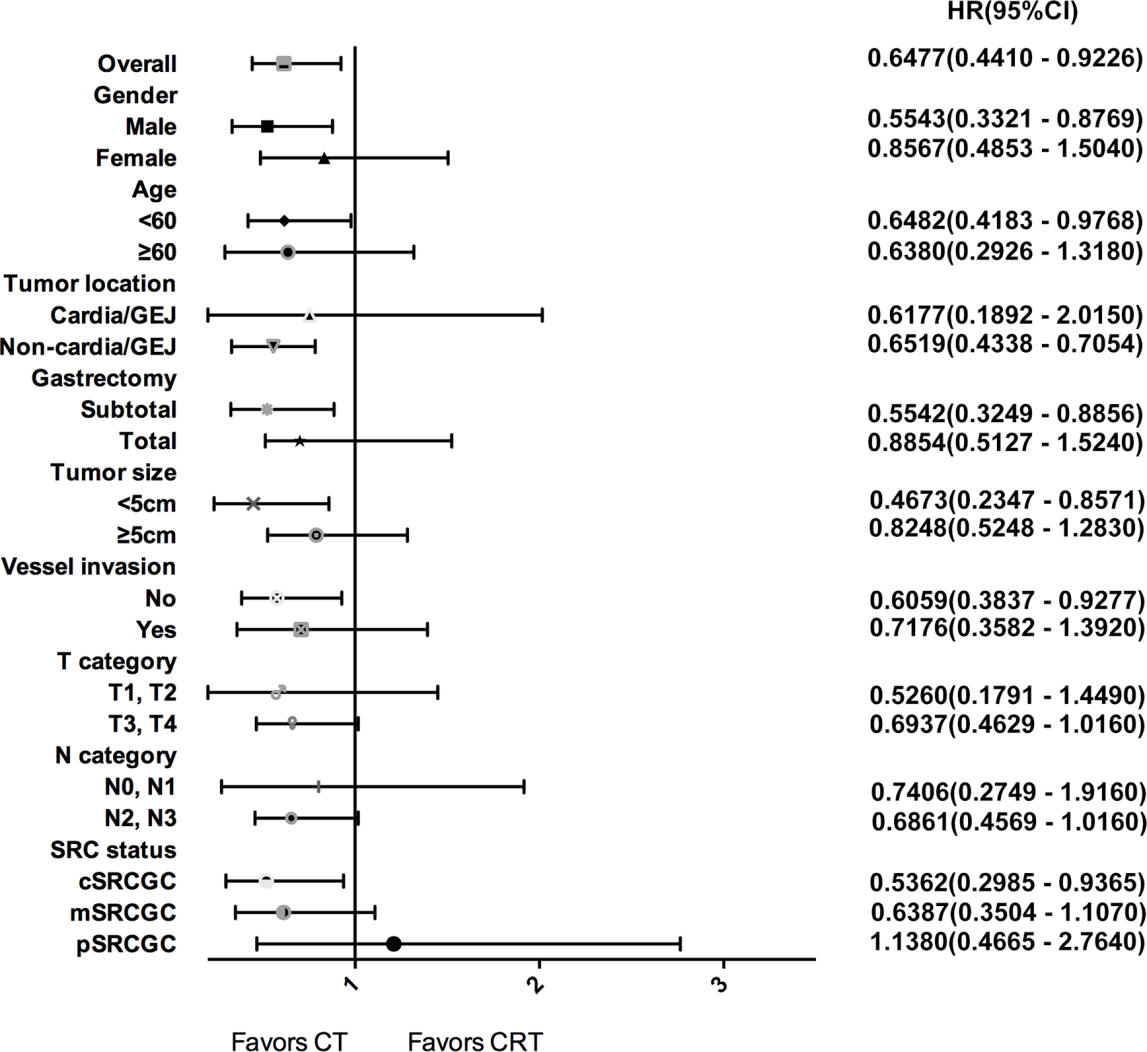
Forest plot for survival hazard ratios and CIs for treatment within subsets of the selected variables.

Subgroup analyses were performed to identify patients who may benefit from chemotherapy (**Figure 3**). Overall, the 3-year OS rate was higher in the CT group than that in the CRT group among all SRCGC patients. For the SRCGC subsets, a higher 3-year OS rate (72.5% vs. 61.1%, *P* = 0.018) in the CT group compared to the CRT group was found particularly in cSRCGC. Additionally, adjuvant chemoradiotherapy obviously weakened survival in SRCGC patients whose lesions were located in the middle-lower third of the stomach (58.3% vs. 70.1%, *P* = 0.026). Meanwhile, another four independent variables were predictive of a poor prognosis in the CRT group: subtotal gastrectomy (62.9% vs.76.0%, *P* = 0.015), male (56.3% vs. 66.7%, *P* = 0.013), <60 year (58.5% vs. 69.7%, *P* = 0.040), and none vessel invasion (61.1% vs. 74.6%, *P* = 0.023).

## Discussion

Despite considerable advances in treatment, the prognosis in GC patients is still poor, especially in cases of diffuse subtype or SRC adenocarcinoma. Adjuvant chemotherapy is a recommendable treatment for resectable GC, with the potential of improving survival outcome (17). The ACTS-GC trial suggested that 1-year adjuvant chemotherapy with S-1 had a better improvement in OS than gastrectomy alone (19). The CLASSIC study found a better DFS with adjuvant chemotherapy, and a capecitabine and oxaliplatin (XELOX) regimen after D2 gastrectomy versus D2 gastrectomy only (18). However, after a curative gastrectomy with D2 lymphadenectomy, the effect of radiotherapy is still controversial.

The ARTIST trial compared the adjuvant capecitabine and cisplatin (XP) regimen to chemoradiotherapy (XP plus radiotherapy with capecitabine) in patients with D2 gastrectomy, while the addition of radiotherapy did not improve the DFS and OS significantly (21). Subgroup analysis showed additional radiotherapy did not improve outcome of patients with diffuse subtype compared to chemotherapy in the Intergroup 0116 (INT-0116) trial (20). Similarly, the ARTIST and CRITICS trials demonstrated that adjuvant chemoradiotherapy did not have additional effects than chemotherapy in the diffuse subtype either (21, 26). Consistent with these findings, our study showed the 3-year OS rate was higher in the CT group compared to the CRT group despite the proportion of SRC. Instead of bringing additional survival benefit in SRCGC, adjuvant chemoradiotherapy might even impair the survival outcome in patients with cSRCGC, a tumor located in the middle-lower gastric, subtotal gastrectomy, male, <60 years old, and no vessel invasion patients. Tumor size ≥ 5cm and the presence of vessel invasion were also the independent prognostic markers for poor prognosis.

Together with our and previous research results, adjuvant chemoradiotherapy might not benefit patients with SRCGC. It may be due to the fact that diffuse gastric cancer appears to have decreased intracellular adhesion as a result of E-cadherin mutation and/or hypermethylation (27), which may further promote the ability of early metastases and to form peritoneal metastases. Our results also indicated the most common recurrence sites was peritoneal metastasis in SRCGCs. As suggested by Brooks et al. (28), if the decreased efficacy of chemoradiotherapy in diffuse subtype is confirmed, future trials may consider different adjuvant approaches based on histology.

Not only is the benefit of additional radiotherapy for SRCGCs still controversial, but there is no currently recognized standard regimen for SRCGCs in adjuvant setting due to poor tumor differentiation and lower chemosensitivity (10, 11). Chen et al. (29) evaluated docetaxel-based and oxaliplatin-based regimens as adjuvant chemotherapy in 991 GC patients. In the pSRCGC subgroup, OS had no significant improvement with chemotherapy against surgery only. However, in the mSRCGC subgroup, those treated with docetaxel-based regimens obtained a better OS, as well as a lower risk of recurrence and cancer-related death compared to oxaliplatin-based regimens. Pernot et al. (30) administrated untreated advanced SRCGC triplet chemotherapy, with docetaxel, 5-fluorouracil, and oxaliplatin (TEFOX). TEFOX appeared to be more effective as first-line treatment in advanced SRCGC. Therefore, regarding ideal regimens for resectable SRC as adjuvant chemotherapy, both oxaliplatin-based and docetaxel-based regimens are the top candidates, and the docetaxel-based regimen may specially benefit mSRCGC. In China, regimens based on docetaxel, oxaliplatin, capecitabine, cisplatin, or 5-fluorouracil, as well as those modifications, were the considered options by Chinese oncologists (29). In our present research, oxaliplatin-based regimes (mFOLFOX6 and SOX) were more often used than docetaxel-based schemes, and no adverse event incidences were found different among those schemes. Instead, the only discrepancy was between CT and CRT. Patients in the CRT group tended to more frequently have leukopenia and neutropenia, which may be explained by concurrent radiotherapy and chemotherapy doing more harm to the hematological system than chemotherapy alone. Further comparison on adjuvant therapy between docetaxel-based and oxaliplatin-based regimen in SRCGC patients after surgical resection would be necessary.

Our study has several limitations. First, the nature of retrospective design without randomized allocation made selection bias unable to be avoided. Patients in the CT group tended to have earlier N category than those in the CRT group. However, the differences between the number of N0 and N3 categories in the CT and CRT groups were not obvious. In multivariate analyses, the N category was not proven to be an independent factor of poor survival, which may have little effect on the final results. Second, similarly due to the retrospective nature, the variation of regimens might introduce potential performance bias. Third, the definite SRCGC only contains pSRCGC (100%) and mSRCGC (>50%) according to WHO classification, thus cSRCGC (≤50%) may partially function as a negative control. In our study, 44.3% of patients were cSRCGC, which may lead to the lower power of definite SRCGC (pSRCGC and mSRCGC) subgroup to gain robust conclusion. Finally, it must be considered that the classification of SRCGC subtypes may differ among pathologists. Nevertheless, there are some advantages we have to mention. To our knowledge, it might be the first data of SRCGC patients comparing adjuvant chemotherapy with chemoradiotherapy.

In conclusion, our study might be the first data of SRCGC patients comparing adjuvant chemotherapy with chemoradiotherapy. Adjuvant chemoradiotherapy may not bring additional survival benefits compared to adjuvant chemotherapy in SRCGC patients with D2 gastrectomy. Specially, chemoradiotherapy should be considered with caution in patients with signet ring cell proportion less than 50%, lower-middle site tumor, partial gastrectomy, male, <60 years old, and have no vessel invasion. Therefore, adjuvant chemoradiotherapy shouldn’t be performed routinely for SRCGC patients in general practice. We suggest that a tailored adjuvant scheme could be further investigated based on SRC status, and high-qualified prospective trials are required to obtain more robust evidence.

## Data Availability Statement

The raw data supporting the conclusions of this article will be made available by the authors, without undue reservation.

## Author Contributions

H-FG, XW, and DC participated in the study design. Y-TZ, X-ZC, and YC participated in manuscript preparation. D-YL, MQ, and QL designed the methodology. YY, Y-LS, and Z-PL participated in data analysis and prepared figures. Y-TZ, Y-WZ, and L-ST collected the detailed information of patients. FB and J-YL reviewed and revised manuscript. All authors contributed to the article and approved the submitted version.

## Funding

The authors disclosed receipt of the following financial support for the research, authorship, and publication of this article: This work was supported by the National Natural Science Foundation of China (Grant No. 81672397).

## Conflict of Interest

The authors declare that the research was conducted in the absence of any commercial or financial relationships that could be construed as a potential conflict of interest.
